# Characterization and comparison of mitogenomes of three ‘eyed’ turtles *Sacalia* spp

**DOI:** 10.1080/23802359.2020.1806750

**Published:** 2020-08-26

**Authors:** Liu Lin, Huaiqing Chen, Zhao Wang, Daniel Gaillard, Xiaofei Zhai, Haitao Shi

**Affiliations:** a Ministry of Education Key Laboratory for Ecology of Tropical Islands, Key Laboratory of Tropical Animal and Plant Ecology of Hainan Province, College of Life Sciences, Hainan Normal University, Haikou, China; b Center for Nature and Society, School of Life Sciences, Peking University, Beijing, China; c School of Science, Technology, and Mathematics, Dalton State College, Dalton, GA, USA

**Keywords:** *Sacalia bealei*, *Sacalia quadriocellata*, *Sacalia insulensis*, mitogenome

## Abstract

In this paper, we characterized the mitogenomes of three ‘eyed’ turtles, the Beal’s Eyed turtle (*Sacalia bealei*), the Four Eye-spotted turtle (*S. quadriocellata*), and the Hainan Four Eye-spotted turtle which was a unique lineage in the four-eyed turtle and considered as an independent species *S. insulensis* recently. The full lengths of the *S. bealei* and *S. quadriocellata* mitogenomes are 16,564 bp, and 16,555 bp, respectively, while the length of partial mitochondrial genome of *S. insulensis* is 16,433 bp without tailed part of D-loop. All the genes exhibit the typical mitochondrial gene arrangement and transcribing directions of turtles. Phylogenetic analysis indicating that a deep divergence about 7.8% p distance was found between *S. bealei* and (*S. quadriocellata + S. insulensis*), and the divergence (2.8% in patristic distance) between *S. quadriocellata* and *S. insulensis* is comparable with other closely related species in turtles.

Turtles of genus *Sacalia* are currently composed of two species: the Beal’s Eyed turtle (*S. bealei)* and the Four Eye-spotted turtle (*S. quadriocellat*a). *Sacalia bealei* is endemic to southern China, while the distribution of *S. quadriocellata* ranges from Vietnam and Laos in southeast Asia to southern China and Hainan island (Turtle Taxonomy Working Group 2017). Due to over-harvesting for pet trade, they have become extremely rare in the wild (Hu et al. 2016; Gong et al. 2017) and listed as endangered on the IUCN Red List (Rhodin et al. 2018) .

*Sacalia quadriocellata* from Hainan island is unique in appearance compared to other populations from mainland China and Southeast Asia. Previous studies based on phylogenetic analysis of CytB gene, and comparative morphological analysis indicated that it should be classified as a valid species named as the Hainan Four Eye-spotted turtle (*S. insulensis*; Shi et al. 2008; Lin et al. 2018). This suggestion has been accepted in the latest updated checklists of reptiles of China (Wang et al. 2020).

In this study, we characterized and compared the complete mitogenomes of the three ‘eyed’ turtles collected from southern China, with the *S. bealei* from Zhangzhou (117.37 E, 24.53 N), Fujian province, the *S. quadriocellata* from Yangjiang (111.59 E, 22.21 N), Guangdong province and the *S. insulensis* from Qiongzhong (109.88E, 19.06N), Hainan island. They were wild caught and then bred alive in captive at Turtles Research and Conservation Center of Hainan Normal University, Hainan Province, China (110.35E, 20.00N). Blood samples were collected from the sub-vertebral site of live turtles following the McArthur et al. (2004) methodology, and stored at this center with code EyedTur01, EyedTur02 and EyedTur03. Genomic DNA was extracted with the DNeasy Blood and Tissue Mini Kit (QIAGEN, Hilden, Germany), following the product protocol. 7.0–7.2 Gb bases were sequenced for each sample, in 150 bp each read, pair end, with the Illumina NovaSeq 6000 System. Raw reads were trimmed for Illumina adapters at right end and low-quality bases at both ends with BBDuk Trimmer 1.0 and mapped to public mitogenome sequences *S. bealei* (GenBank Accession Number GU183364) and *S. quadriocellata* (GU320209), with Low Sensitivity/Fastest, Fine Tuning(None), in Geneious Prime^®^ 2020.0.3. 14,296, 11,303 and 1820 reads were matched to the reference respectively for *S. bealei*, *S. quadriocellata* and *S. insulensis*, generating contigs with an average coverage at 128.4, 101.6 and 16.4-fold. Consensus sequences were generated for bases covered with at least 2 reads and annotated according to the references with the *Transfer Annotations* function in Geneious Prime^®^ 2020.0.3, which were accessible in GenBank with accession numbers MT372820–MT372822.

The length of complete mitochondrial genome of *S. bealei* and *S. quadriocellata* is 16,564 bp and 16,555 bp, and the length of partial mitochondrial genome of *S. insulensis* is 16,433 bp without tailed part of D-loop. The structure of mitochondrial genomes of the three ‘eyed’ turtles are similar to other turtle species, including 13 protein coding genes, 2 rRNA genes, 22 tRNA genes, and 1 control region, whose base composition is 34% A, 26% C, 13% G, 27% T, and 39% GC. Most genes are coded in heavy strand except *tRNA-Gln*, *tRNA-Ala*, *tRNA-Asn*, *tRNA-Cys*, *tRNA-Tyr*, *tRNA-Ser*, *tRNA-Glu*, *tRNA-Pro* and *ND6* gene. Most protein-coding genes use common start codon ATG and stop codon TAA, except rare start codon GTG and rare stop codon AGG in *CO1* gene. It is common that TAA stop codon is completed by the addition of 3′-A residues to the mRNA.

Mitogenome sequences of closely related species were aligned with our sequences by Mafft Plugin 1.4.0 installed in Geneious Prime. The phylogeny was inferred from 15,590 bp coding regions excluding the D-loop and nearby tRNA-Phe, using the Bayesian inference in MrBayes plugin 2.2.3 (Huelsenbeck and Ronquist 2001; Ronquist and Huelsenbeck 2003) installed in Geneious Prime, with GTR + I + G suggested by Modeltest (Posada and Crandall 1998), and 1,100,000 iterations with beginning 100,000 burn-in were performed.

In Figure 1, all sequences from Genus *Sacalia* were clustered into a monophyletic group located in the Family Geoemydidae. A deep divergence about 7.8% p distance was found between *S. bealei* and (*S. quadriocellata + S. insulensis*). There is perhaps an identification error that sequence EF088646 is labeled as *S. quadriocellata* in GenBank but is almost exactly the same as the two sequences from *S. bealei,* sharing more than 99% identical sites each other. The divergence between *S. quadriocellata* and *S. insulensis* is about 2.8% in patristic distance, comparable to that between closely related species in genus *Cuora*, such as 3.0% between *C. trifaciata* and *C. pani*, and 3.8% between *C. galbinifrons* and *C. bourreti*. The results obtained here can contribute to phylogenetic analysis and biological conservation of ‘eyed’ turtles further.

**Figure 1.**
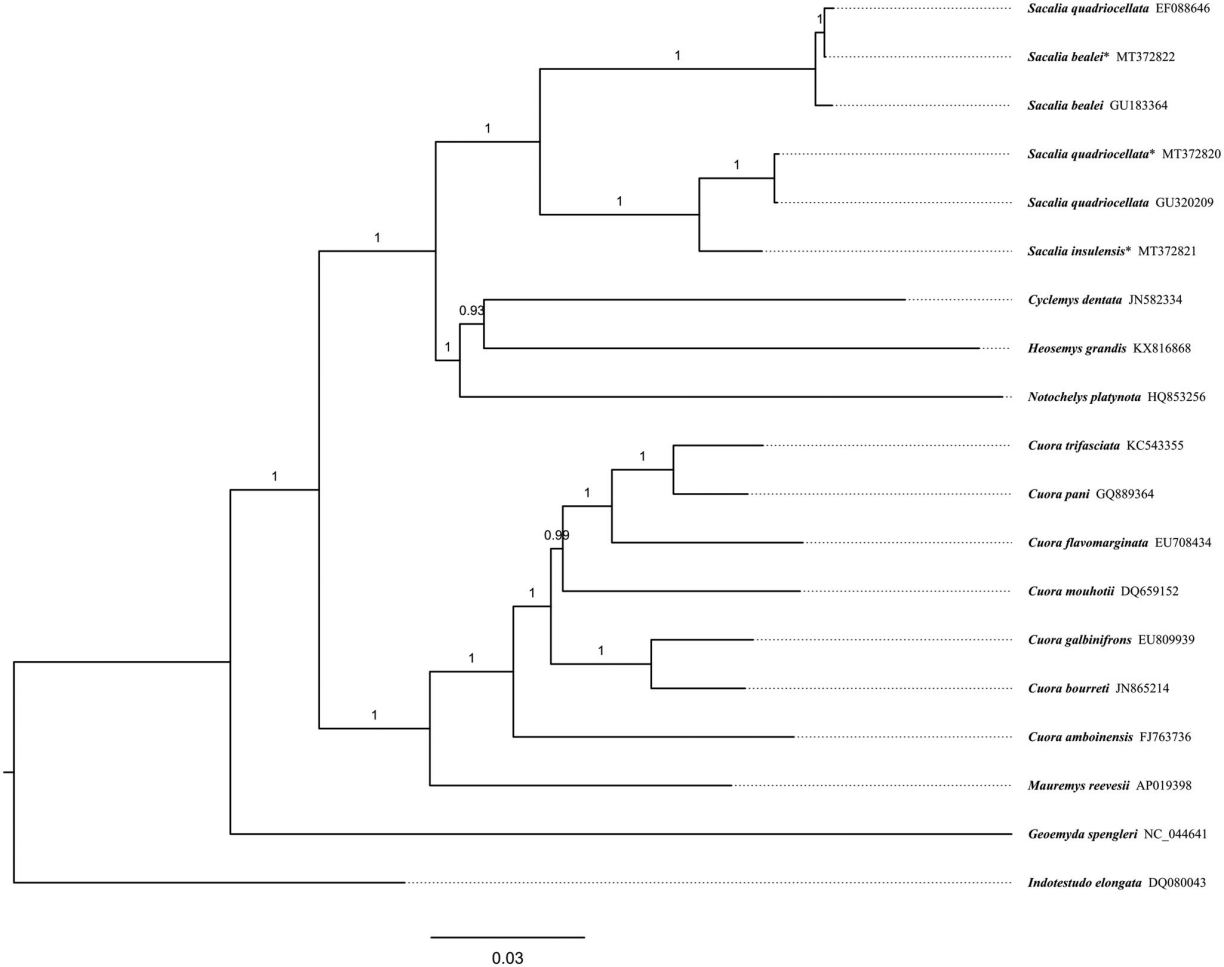
Bayesian tree of ‘eyed’ turtles *Sacalia* spp. based on mitogenomes excluding D-loop and nearby tRNA-Phe. *Indotestudo elongata* (DQ080043) was set as an outgroup. Posterior probability values are shown at branches. Scientific names and GenBank accessions number are labeled at tips. Mitogenomes sequenced in this study are marked with an asterisk.

## Data Availability

The data that support the findings of this study are openly available in GenBank of NCBI at https://www.ncbi.nlm.nih.gov, reference number MT372820, MT372821 and MT372822.
